# Pozzolanic Reactivity of Silica Fume and Ground Rice Husk Ash as Reactive Silica in a Cementitious System: A Comparative Study

**DOI:** 10.3390/ma9030146

**Published:** 2016-03-01

**Authors:** Weiting Xu, Tommy Yiu Lo, Weilun Wang, Dong Ouyang, Penggang Wang, Feng Xing

**Affiliations:** 1 Guangdong Provincial Key Laboratory of Durability for Marine Civil Engineering, College of Civil Engineering, Shenzhen University, Shenzhen 518060, China; xingf@szu.edu.cn; 2 Department of Architectural and Civil Engineering, City University of Hong Kong, Hong Kong, China; bctommyl@cityu.edu.hk; 3 Department of Mechanics and Civil Engineering, College of Science and Engineering, Jinan University, Guangzhou 510632, China; toud@jnu.edu.cn; 4 Ceneter for Durability and Sustainability Studies of Shandong Province, Qingdao Uiniversity of Technology, Qingdao 266033, China; wangpenggang007@gmail.com

**Keywords:** silica fume, rice husk ash, pozzolanic reactivity, strength, chloride ion penetration resistance, morphology

## Abstract

This study comparably assessed the pozzolanic effect of silica fume (SF) and ground rice husk ash (RHA) as supplementary cementing materials on the properties of blended cement pastes and concretes. A commonly commercial silica fume (SF) and locally-produced rice husk ash (RHA) samples with two finenesses (one with larger size than cement and the other with smaller size than cement) were used in this study. Material properties of SF and RHA were experimentally characterized. Hydration and mechanical properties of cement pastes incorporating SF and RHA were determined by thermogravimetric analysis (TGA) and compressive strength tests, respectively. Properties of concretes regarding workability, mechanical property, durability, and microstructure were evaluated. Results showed that, although the finely ground RHA used in this study possessed lower SiO_2_ content and higher particle size compared to SF, it exhibited comparable pozzolanic reactivity with SF due to the nano-scale pores on its each single particle, leading to a higher specific surface area. The optimal replacement levels of SF and RHA were 10% by weight of cement in pastes and concretes. Although addition of SF and RHA led to a significant reduction in slump for the fresh mixtures, inclusion of up to 30% of SF or 15% of ground RHA did not adversely affect the strength of concretes. At the same mix, incorporation of finely-ground RHA in cement composites provided comparable mechanical properties, hydration degree, and durability with SF blended cement composites, owing to the porous structure and high specific surface area of RHA particles. Microstructure morphology analysis of concretes explored by scanning electron microscopy (SEM) further validated the strength and the durability test results.

## 1. Introduction

In the manufacture of cement, the clinker production process requires a great amount of energy and emits a large amount of carbon dioxide (CO_2_) into the atmosphere. The increase in CO_2_ emissions has led to the greenhouse effect and an increase in the temperature of the Earth. To reduce the environmental problems, industrial and agricultural byproducts such as fly ash, metakaolin, granulated blast furnace slag, *etc.,* have been used as supplementary cementing materials to reduce the production of cement, thus reducing the emission of CO_2_ and the use of energy [1,2,3,4,5,6,7]. Moreover, the incorporation of these cement replacement materials in concretes has been reported to improve the mechanical properties and penetration resistance of the concretes [8,9,10,11,12,13].

Silica fume (SF) and rice hush ash (RHA) are both siliceous materials. They are highly concentrated sources of amorphous SiO_2_ and possess similar chemical reaction mechanism in cement matrix system. The reactivity of SF and RHA contributes to the strength enhancement of cement-basted materials by pozzolanic reactions between amorphous silica and calcium hydroxide liberated during the cement hydration process. These reactions produce additional amounts of C-S-H gel that makes denser microstructure of SF and RHA containing cement-based materials [14,15].

SF is very fine non-crystalline silica produced by an electric arc furnace as a byproduct of the smelting process in the production of metallic silicon or ferrosilicon in the alloys industry. The reduction of high-purity quartz to silicon at temperatures up to 2000 °C produces SiO_2_ vapors, which oxidize and condense in the low-temperature zone to tiny particles consisting of 85%–99% amorphous silica [16]. Then, SF is composed of submicron particles of silicon dioxide, which occur as almost-perfect spheres with diameters ranging from 20 to 500 nm [17]. It is estimated that current global output of SF is, at most, between one and 1.5 million tonnes per year [18]. It has been widely reported that higher strength and durability enhancement can be obtained for concretes containing silica fume. Performance of silica fume concretes in sulfate environment is also better than normal Portland cement concretes [19,20,21].

RHA is the combustion residue of the rice husks, which are the shells produced during the dehusking operation of paddy rice. Each tonne of paddy rice produces about 200 kg of husk which, on combustion, yield approximately 40 kg ash. Rice plants ingest orthosilicic acid from groundwater, whereupon it is polymerized to form amorphous silica in the husks [22]. After combustion, the organic compounds are devolatilized as carbon dioxide, while the silica is retained in the ash residue. Studies have shown that the main chemical composition of RHA is biomass silicon dioxide (SiO_2_), and processing conditions have a significant effect on the characteristics of silica in RHA. The ash formed during open-field burning or uncontrolled combustion in furnaces generally contains a large proportion of less reactive crystalline silica minerals such as cristobalite and tridymite. The highest amount of amorphous silica is achieved when RHA is burned between 500 and 700 °C [23]. Reactivity of RHA is attributed to its high content of amorphous silica and its very large surface area governed by the porous structure of the particles [24]. Generally, the reactivity is also favored by increasing the fineness of the ash [25]. As a pozzolanic material, RHA can be used to replace Portland cement by up to 15% [26]. RHA with high fineness can improve the compressive strength and produce mortars with low porosity. For durability, the use of RHA significantly improves resistance to water permeability, chloride ion penetration, and sulfate deterioration of concretes [27,28,29].

To date, intensive research efforts have been made in optimal cement replacement level and enhancement of blended concretes for SF dust and RHA. It has been widely accepted that SF and RHA as super pozzolans can significantly improve various properties of concretes. However, it is not aware of any literature wherein a systematically comparative study on pozzolanic reactivity of SF and ground RHA as reactive silica in cementitious system. Moreover, one of the main dimensions of this investigation was to explore the feasibility of RHA instead of SF (which is not economically available even in developed countries) in mortars and concretes. This would also reduce the environmental problems associated with the current open field burning of the rice husk. Therefore, efforts may be made to encourage the use of RHA in construction materials.

This paper launches a comparative study to assess the pozzolanic reactivity of SF and RHA. The hydration properties of cement pastes incorporating SF and RHA after the compressive strength test were determined by thermogravimetric analysis. Properties regarding workability and mechanical properties of concretes were monitored by slump test, compressive strength test, and splitting tensile strength test. The durabilities of concretes were evaluated by rapid chloride iron penetration testing. The microstructures of concretes were explored through scanning electron microscopy (SEM) analysis.

## 2. Experimental

### 2.1. Constituent Materials of Blended Cement Pastes and Concretes

An ordinary Portland cement provided from Green Island Cement Co Ltd (Hong Kong) CEM II 52.5 complying with BS12 [30] standard was used. The cement had a specific gravity of 3.12 and the fineness of 0.34 m^2^/g.

A commonly commercial silica fume used in this study was supplied from Xilong Chemical Co., Ltd. (Shantou, China).

Locally-produced RHA samples were obtained by burning rice husk at a specified temperature of 600 °C for two hours at a heating rate of 20 °C/min in an electronic furnace. For the grinding process, a laboratory stainless ball mill manufactured by Nanjing University, Nanjing, China, was used to grind the raw ash to smaller particles. The rotational milling speed was set as 1000 RPM, and the mass ratio with ball to powder was 20:1. The grinding duration was set as 5 and 30 min, respectively. After each grinding intermission the ground sample was taken out to keep in a dry sealed container for subsequent tests.

River sand with a fineness modulus of 2.44 and specific gravity of 2.65 was used as the fine aggregate. Crushed granites with two size ranges (20 and 40 mm) and having specific gravity of 2.67 were used as the coarse aggregate.

A naphthalene-based superplasticizer, Daracem^@^108, Grace Construction Products, Hong Kong, produced in liquid form was used.

### 2.2. Characterization of Constituent Materials

The chemical compositions of constituent materials were monitored by X-ray fluorescence (XRF). Mineralogical analyses were performed by X-ray diffractometry (XRD), using a Phillips PW 1050 diffractometer (Philips, Belgrade, Serbia) with a 50 kV, 50 mA Cu radiation source. Particle morphologies were taken by environmental scanning electron microscope (ESEM) (FEI XL30 S-FEG, FEI Co., Hillsboro, OR, USA) with a 20 kV accelerating voltage. Mean particle sizes were monitored by a laser particle size analyzer (Microtrac SR150, Microtrac Inc., Dallas, TX, USA). Specific gravities were measured according to ASTM D5550-14 [31]. Specific surface areas were determined by the Blain method for cement and nitrogen adsorption analysis for SF and RHA.

### 2.3. Preparation and Testing of Blended Cement Pastes

SF and RHA blended cements were prepared by replacing 10% of cement weight by ground RHAs and SF in a dry condition. The mixtures were thoroughly homogenized and kept in sealed bags. The blended cement pastes were prepared with a water to binder ratio of 0.4 at room temperature of 20 ± 2 °C. All of the fresh paste mixtures were cast into 20 mm × 20 mm × 20 mm cube molds. After 24 h, the pastes were demolded and cured at 20°C with RH higher than 95% until the day for the subsequent tests.

As for the compressive strength tests, all the cementitious pastes were tested at 1, 3, 7, 28, and 90 days in accordance with ASTM C109 standard [32].

In order to study the hydration reaction of the cementitious pastes, the paste specimens were ground to obtain homogeneity of the grain size below 50 microns and subjected to the quantitative thermogravimetric analysis (TGA). The TGA heating temperature range was run at 50–900 °C and with a heating rate of 10 °C per minute. The TGA curves were plotted and the weight losses of the pastes at 1, 3, 7, and 28 days were calculated. As the different chemical compounds in cement correspond to certain decomposition weight losses of TGA curves, the qualitative evaluation of the hydration degree of pastes can be assessed [33].

### 2.4. Mix Proportions of Concretes

Concrete mix proportions were listed in Table 1. 13 concrete mix proportions were prepared with the same amount of binder (560 kg/m^3^) and water to binder ratio (w/b) of 0.3.

Cement was partially replaced by SF and finely ground RHA (FRHA) at 5%, 10%, 15%, 20%, 25%, and 30% by mass. The amounts of superplasticizer in all concrete mixtures were adjusted in order to control the slump of fresh control concrete between 60 and 90 mm.

### 2.5. Preparation and Testing of Concretes

The effects of SF and ground RHA on the workability of concrete were determined by comparing slump values of fresh concretes.

The compressive strength test was carried out in concrete cubes of size 100 mm × 100 mm × 100 mm with w/c ratio of 0.3. After water curing for 7, 14, 28, and 90 days, the specimens were subjected to compressive strength tests using an AIMIL compression testing machine (Aimil Ltd., New Delhi, India) of 2000 kN capacity at a rate of loading of 140 kN/min. The tests were carried out on triplicate specimens and the average compressive strength values were recorded.

The splitting tensile test was carried out in concrete cylinders of size 100 mm diameter and 200 mm height as per ASTM C496 [34]. After water curing for 28 days, the concrete cylinders were subjected to splitting tensile test by using a universal testing machine. Tests were carried out on triplicate specimens and the average splitting tensile strength values were recorded.

The durability of concretes was determined by the rapid chloride ion penetration test (RCPT) as per ASTM C1202 [35]. Concrete discs with 85 mm diameter and 50 mm thickness were cast and cured for 28 days. After curing, the concrete specimens were subjected to RCPT test by impressing 60 V. Two halves of the specimen was sealed with PVC container of diameter 90 mm. One side of the container is filled with 3% NaCl solution (that side of the cell will be connected to the negative terminal of the power supply), the other side is filled with 0.3 N NaOH solution (which will be connected to the positive terminal of the power supply). Current is measured every 30 min for up to 6 h. Chloride contamination and temperature at every 30 min was also monitored. From the results using current and time, chloride permeability was calculated in terms of Coulombs at the end of 6 h.

The morphology of concretes after curing for 90 days was determined by a JSM 820 scanning electron microscope (SEM, JEOL, Tokyo, Japan) with an acceleration voltage of 15–20 keV.

## 3. Results and Discussion

### 3.1. Chemical Compositions and Physical Properties of SF and RHA

**Figure 1:**
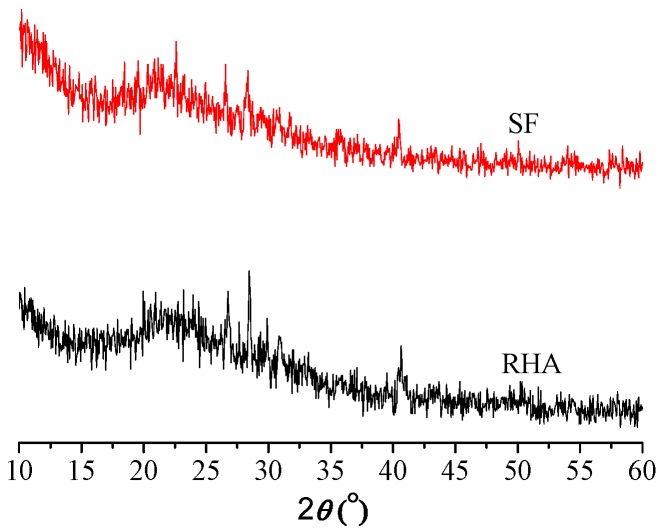
XRD spectrum of SF and RHA.

The oxide analyses for cement, SF, and RHA samples are listed in Table 2. Table 2 shows a significantly high content of amorphous SiO_2_ in SF, with small amounts of iron, magnesium, alumina, calcium, and alkali oxides. RHA exhibits a lower SiO_2_ amount (82.9%) than SF (94%). XRD analysis (Figure 1) showed that SF and RHA possess similar mineralogical spectra, and their silica phases corresponding to 22.5° (2θ) were mostly in amorphous form, which are both active.

**Figure 2:**
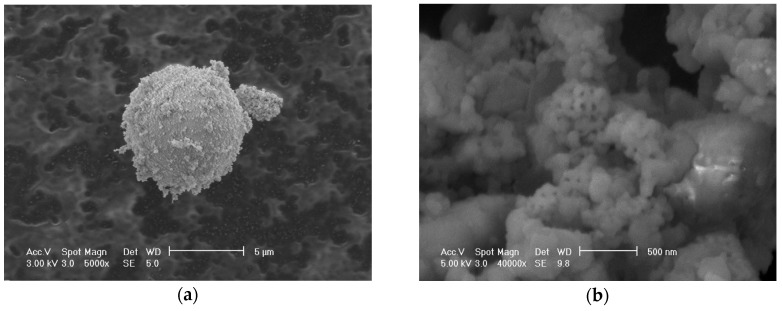
SEM images of SF and ground RHA: (**a**) SF; (**b**) FRHA (30 min grinding).

The physical properties of cement, SF, and RHA are recorded in Table 3. It is seen that specific gravity of SF and RHA are less than that of cement. The specific gravity of RHA increase with the increase of grinding duration. The mean particle size of RHA decreases from 9.49 to 5.69 *μ*m with a grinding duration from 5 to 30 min and, accordingly, the specific surface area rises from 19.4 to 23.6 m^2^/g. The mean particle size of FRHA (d_50_ = 5.69 *μ*m) is larger than that of SF (d_50_ = 5.11 *μ*m). However, FRHA presents a higher specific surface area compared to SF, attributed to its very porous structure of each single particle. This analysis is complemented with the SEM images in Figure 2. As shown in Figure 2a, spherical particles of silica fume present a smooth, dense surface. The FRHA powder surface is very porous as seen in Figure 2b. The size of these surface pores on FRHA particles is less than 50 nm. These nano-scale pores greatly contribute to high specific surface area and high pozzolanic reactivity of RHA.

### 3.2. Compressive Strength of Blended Cement Pastes

**Figure 3:**
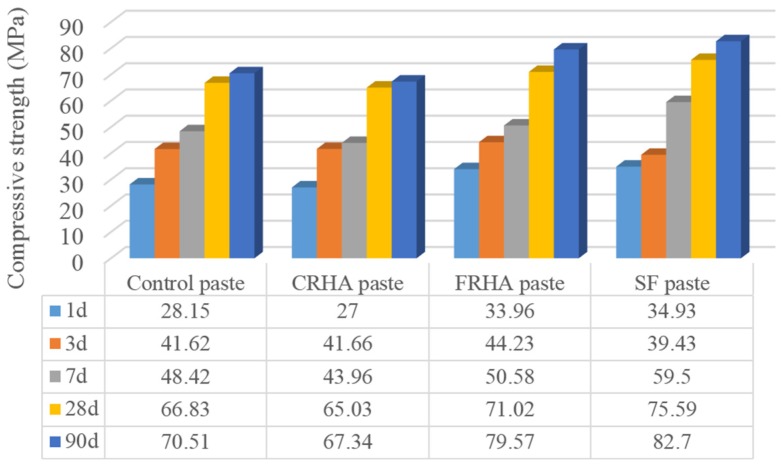
Compressive strength of pastes.

The compressive strength results of cement paste and pastes with incorporation of 10% cement replaced by CRHA, FRHA, and SF is shown in Figure 3. The SF blended paste shows the highest strength value (82.70 MPa) among all the pastes at the age of 90 days, which is 17% higher than the control paste. This is related to the high content and high surface area of pure glassy silica in SF, which exhibits excellent pozzolanic reactivity and the well packing effect of SF particles in the cement composite.

It can be seen that the paste incorporating CRHA shows the lowest compressive strength at all the testing curing ages, which may be due to the larger particle size and lower surface area of coarse RHA particles. Strength of paste incorporating RHA increases with increase of grinding duration from 5 to 30 min. The FRHA paste presents a 79.59 MPa compressive strength at the age of 90 days, which is 12.8% higher than that of the control paste and exhibits comparable strength value with SF paste (82.70 Mpa). Results show that the addition of finely ground RHA to paste gives rise to an increase of the compressive strength compared to the control concrete, due to the increasing specific surface area and pozzolanic reactivity of RHA.

### 3.3. Hydration Process of Blended Cement Pastes at 1, 7, and 28 Days

**Figure 4:**
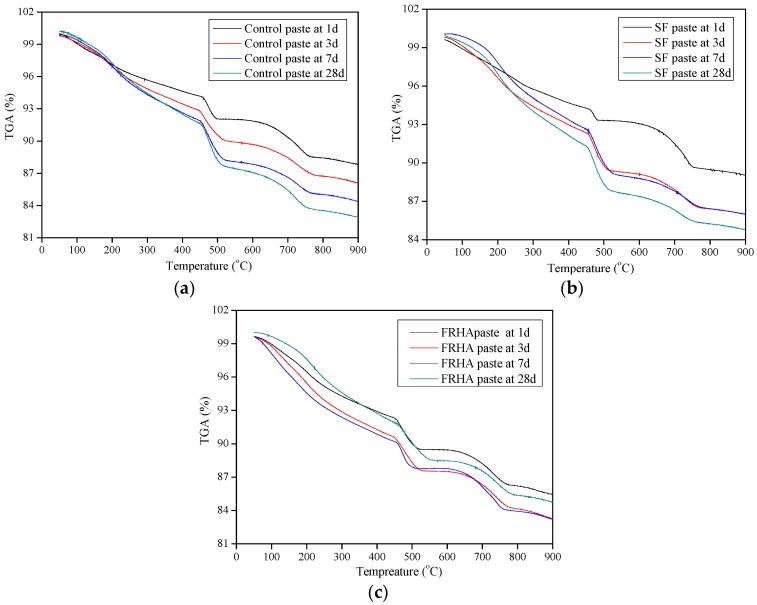
(**a**) TGA curve of control paste at 1, 3, 7, and 28 days; (**b**) TGA curve of SF paste at 1, 3, 7, and 28 days; and (**c**) TGA curve of FRHA paste at 1, 3, 7, and 28 days.

The thermogravimetric weight losses of control, SF paste and FRHA paste samples at 1, 7, and 28 days were plotted in Figure 4a–c. It is seen that curves of all pastes show three rapid weight losses. The first weight loss, located between 110 and 300 °C, is mainly due to dehydration of C-S-H. The second major weight loss, observed at 450–550 °C, corresponds to the dehydroxylation of portlandite, another hydration product. The third weight loss appears at 750 °C, which corresponds to the decarbonation of calcium carbonate deriving from the cement clinker [33]. The quantitative analysis for the first weight loss and the second weight loss can be used as an indicator of the hydration reaction degree of the cement composite matrix.

The weight losses of paste specimens are summarized in Table 4. These values are calculated from the testing data in Figure 4. It is observed that the weight loss of the control paste is increased from 40.57% to 44.70% with an increase in the curing age from 1 to 7 days. The weight loss of the control paste is 44.69% at the age of 28 days, indicating the weight loss for the control paste remains even at the curing age from 7 to 28 days. The weight loss of SF at the first stage is increased from 46.59% to 51.04% at the curing age from 1 to 28 days. For the FRHA paste, the weight loss caused by the dehydration reaction at the first stage is increased from 44.21% to 49.90% with increase in curing age from 1 to 28 days. Comparing the dehydration weight losses of all the paste samples at the age of 28 days, the SF presents the highest value compared to the control paste and the FRHA paste. The weight loss of dehydration of the FRHA paste follows the SF paste by second and shows 5.12% higher than that of the control paste. Results also indicate that the rate of hydration of FRHA paste is initially lower than that of SF paste but higher than the control paste. Therefore, SF particles can contribute to the high early age strength and the FRHA increases the long-term strength, although this strength is lower than SF blended paste at all ages, which is consistent with previous reports [20].

As for the second weight loss stage corresponding to dehydroxylation, the control, the SF paste and the FRHA paste shows 33.85%, 28.67%, and 31.04%, respectively, at the age of 28 days. The SF paste and the FRHA paste show lower weight loss percentage in dehydroxylation reaction corresponding to Ca(OH)_2_ dehydroxylation compared to the control paste, which also indicates the excellent pozzolanic activity of SF and FRHA.

### 3.4. Workability of Concretes

The slump testing results of fresh concrete mixtures are listed in Table 5. It is observed that the slump of the control concrete is 85 mm. SF addition decreases the slump values of fresh concrete mixtures with increase in 5% to 30% cement replacement. The values of the SF mixtures vary between 95 and 25 mm with an increase in the dosage of SF. With the addition of 5% and 10% cement replaced by FRHA, the slump of the concrete mixtures is 125 mm and 100 mm, and are higher than that of the control concrete mixtures, which may be related to the well packing of the cementitious system. However, the slump value decreases with the increase of FRHA dosage in the mixtures. The slump value is significantly reduced to 15 mm with 25% and 30% cement replaced by FRHA. There are significant declines of slump with increase of SF and FRHA addition in the mixtures with 5% to 30% cement replacement ratio. This may be due to that addition of SF and FRHA aggravates narrow particle size distributions of the concrete mixtures and, hence, resulting in a higher water demand.

### 3.5. Compressive Strength of Concretes

The compressive strength results of concrete specimens are shown in Table 5. It is seen that the compressive strength of the control concrete is 86.81 and 92.52 MPa at 28 and 90 days, respectively.

For series of SF concretes, the 28-day compressive strength of 5%SF, 10%SF, and 15%SF concrete is 91.33, 105.82, and 101.62 MPa, which is 105%, 122%, and 117% of the control concrete, respectively. However, further increasing in cement replacement ratio of SF, the compressive strength of concrete is reduced. At 90 days, the compressive strength of 5%SF, 10%SF, and 15%SF concretes is 98.56, 109.69, and 103.22 MPa, respectively. The highest compressive strength value appears on the 10%SF concrete, which was 119% of the control concrete at the age of 90 days.

Compressive strength of 5%FRHA, 10%FRHA, and 15%FRHA concretes at 28 days is 93.62, 101.97, and 97 MPa or 108%, 117%, and 112% of the control concrete, respectively. At the later age, their strengths are slightly increased, and, 90-day compressive strength of these concretes is 99.51, 106.88, and 102.19 MPa or 108%, 116%, and 110% of the control concrete, respectively. It is observed that the compressive strength of 20%FRHA concrete at 90 days is slightly lower than that of control concrete. Again, increasing the cement replacement ratio of FRHA to 25% and 30% decreases the strength of concrete. Therefore, the optimal ratio of cement replaced by FRHA is 10%. However, the normalized compressive strength of all FRHA concretes increased with the ages. This suggests that the contribution of compressive strength gain is due to the pozzolanic reaction of FRHA with Ca(OH)_2_ released from hydration of cement.

Comparing the compressive strength of the SF concrete and the FRHA concrete at different ages, the incorporation of up to 30% of SF or 15% of FRHA does not adversely affect the strength of cement concrete. A further increase in the cement replacement ratios to 20% FRHA and 25% SF, however, decreases the strength of concrete. For the same replacement ratio of 10%, the strengths of SF and FRHA concretes were similar to each other and significantly higher than that of control concrete. This suggests an excellent pozzolanic reactivity and filler effect of SF and FRHA which makes concrete denser. In addition, the strengths of the SF concretes are higher than those of the FRHA concretes with the same cement replacement ratio, indicating that SF is more reactive than FRHA. This is because the RHA used in this study contains certain amount of unburnt carbon particles, which impairs the purity of the amorphous SiO_2_ and, hence, compromising its pozzolanic activity. To overcome this, satisfactory treatment conditions such as acid leaching of rice husks prior to combustion or mechanical ultrafine grinding of RHA may minimize the effect of the residual carbon or presence of the crystalline compounds.

### 3.6. Splitting Tensile Strength of Concretes

The splitting tensile strength results of SF and FRHA concrete at the curing age of 28 days are shown in Table 5. It is seen that, all the SF concretes exhibit higher splitting tensile strength values than the FRHA concrete at the same cement replacement level. For the FRHA concrete series, at 5% and 10% cement replacement level, the splitting tensile strength of the concrete increases to 63.39 MPa and 68.09 MPa, which is 7.6% and 15.5% higher than that of control concrete, respectively; while beyond 10% cement replacement level by FRHA, the decrease in splitting tensile strength is observed.

### 3.7. Rapid Chloride Permeability of Concretes

The limit values along with classification ratings of chloride penetrability of concrete in accordance with ASTM C1202 [35] are shown at Table 6. The changes of total charge passed of concrete specimens at the age of 28 days are shown at Table 7. The test results in Table 7 have been evaluated according to limit values according to Table 6.

It is seen that the chloride permeability of the control and the SF concretes are evaluated as “very low”. It is found that chloride permeability of FRHA series is “very low” when FRHA is used at 5% to 20% cement replacement ratio. When FRHA is used at 25% and 30% ratios, chloride permeability of concrete rises to “low” and “moderate” level, respectively. It is also found that comparing to the improvement of compressive strength of FRHA blended concretes with optimum replacement levels less than 15% by weight of cement, the chloride permeability of FRHA concretes show improvement of durability with higher optimum replacement dosages up to 20%, which is also in line with previous studies [36,37].

### 3.8. SEM Morphology of Concretes

**Figure 5:**
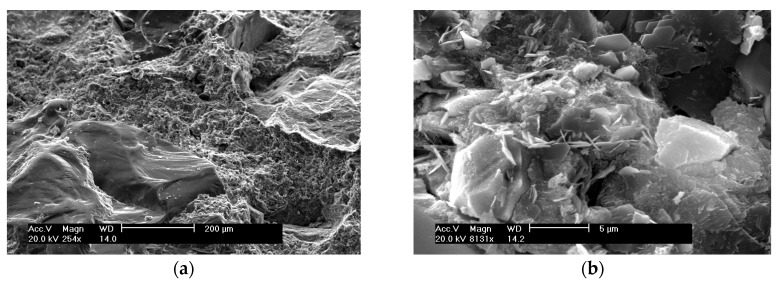
SEM of control concrete: (**a**) with magnification of ×254; (**b**) with magnification of ×8131.

**Figure 6:**
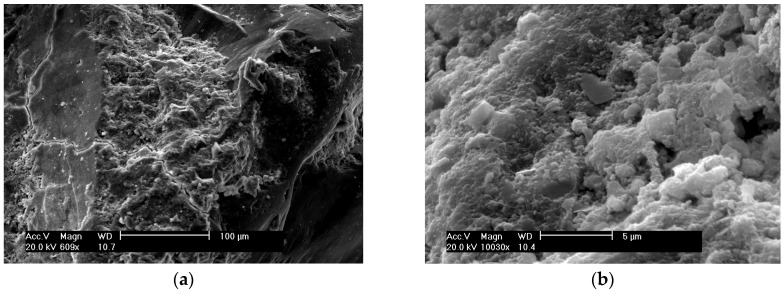
SEM of SF concrete: (**a**) with magnification of ×609; (**b**) with magnification of ×10030.

**Figure 7:**
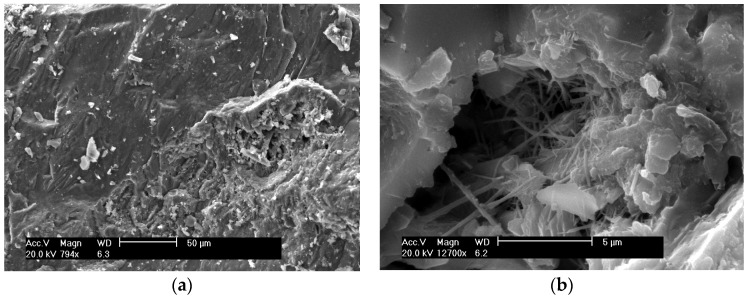
SEM of FRHA concrete: (**a**) with magnification of ×794; (**b**) with magnification of ×12700.

The SEM morphologies of control, SF, and FRHA concrete are shown in Figure 5, Figure 6 and Figure 7. The SEM images of the control concrete are shown in Figure 5a,b. The image in Figure 5a shows a large aggregate on the left side covering the area in the image almost diagonally. The surface is wavy and contains crests and troughs, or concave and convex areas. The adjacent areas are covered loosely porous C-S-H gel. Further magnifying the C-S-H gel area at higher magnifications of ×8131 as shown in Figure 5b, it is seen the C-S-H gel area appears to be dense along with some pores with length of 1 to above 5 μm. A slice of flake-like calcium hydroxide and needle-like crystals are found to cover the surface of C-S-H gel matrix.

Figure 6a presents the morphology of the SF concrete at the age of 28 days. The hydrated cement matrix area at magnification of ×10030 as shown in Figure 6b presents a tightly-packed hydrated C-S-H gel only alone with a few pores with a length of 1 to 3μm, and there is no sign of calcium hydroxide and needle-like crystals. The dense hydrated area of SF concrete indicates a very compact texture.

Figure 7a shows the morphology of fractured surfaces of FRHA concrete curing at room temperature for 28 days. It is seen the cement paste penetrates into the aggregate and forms a stronger bond. Further magnifying the cement hydrate area as shown in Figure 7b, two distinct phases can be clearly seen: the C-S-H gel and the needle-like crystals. The flake-shaped calcium hydroxide crystalline phase does not appear on the glassy phase surface of SF and FRHA concrete, indicating reactive silica in FRHA and SF can enhance and accelerate secondary hydration reaction of Ca(OH)_2_ in cement matrix. However, the needle-like crystalline phase presented in FRHA is possibly due to minor crystallization of the aluminosilicate species, which, to some extent, impairs the strength gain of cement composite. These morphological differences may also be responsible for compressive strength differences among the concrete specimens.

## 4. Conclusions

The following conclusions can be drawn from the results of this study:

1. Although RHA used in this study possesses lower SiO_2_ content and larger particle size compared to SF, the finely-ground RHA presents a higher surface area due to the nano-scale pores on each single RHA particle. These nano-scale pores with size less than 50 nm greatly contribute to the high surface area and high pozzolanic reactivity of RHA.
2. According to the compressive strength results of pastes, at 10% cement replacement ratio, the SF blended paste shows the highest compressive strength value among all the paste samples at the age of 90 days, which is 17% higher than control paste. As for the series of RHA paste, results show that the pozzolanic reactivity of RHA increases with the increase of grinding fineness. FRHA paste presents 12.8% higher than that of the control paste and exhibits comparable strength value with SF paste. The TGA analysis results of paste reveal that the FRHA shows a high pozzolanic reactivity and can be comparable with SF.
3. The concrete tests show that although the optimal level of SF and FRHA content were achieved with 10% cement replacement by mass, test results show a significant reduction in slump for the blended fresh mixtures. It is found that cement could be advantageously replaced by SF and RHA up to maximum limit of 30% and 15%, respectively. At the same mix, SF concrete specimens present higher compressive and splitting tensile strength compared to the FRHA concrete specimens, indicating SF is more reactive than FRHA. This may be attributed to the limitation of burning and grinding treatment condition of RHA in this study. However, superior highly-reactive RHA could be expected if satisfactory pretreatment conditions such as acid leaching of rice husks or mechanical ultrafine grinding of RHA are utilized, which may minimize the effect of the residual carbon or presence of the crystalline compounds in RHA.
4. The chloride permeability of SF blended concretes is “very low” rating when SF content is up to 30%. The “very low” rating of FRHA concretes appears at concrete with FRHA addition within the 20% cement replacement level.
5. The SEM morphology analyses manifest that cement paste penetrates into the aggregate and forms a strong interfacial bond, and there is no sign of calcium hydroxide in SF and FRHA concrete, indicating reactive silica in FRHA and SF can enhance and accelerate the secondary hydration reaction of Ca(OH)_2_ in cement matrix. However, a slice of needle-like crystalline phase presented in FRHA is possibly due to minor crystallization of the aluminosilicate species, which, to some extent, impairs the strength gain of RHA blended cement composite. These morphological differences may also be responsible for compressive strength differences among the concrete specimens.

Based on this experimental study, it is found that finely ground RHA exhibits comparable pozzolanic reactivity with SF and can contribute to various improvements in the performance of cementitious system. Considering SF is not economically available even in developed countries, utilization of RHA in cementitious materials can not only reduce materials costs, but also lessen the environmental burdens associated with the current open field burning of the husk in rice-producing countries. Thus, broad application prospect of RHA instead of SF in cementitious materials could be expected due to the advantages of cost savings, and environmental benefits related to the disposal of waste materials and to reduced carbon dioxide emissions.

## Figures and Tables

**Table 1:** Mix proportions of the concrete specimens.

Concrete ID	w/b	Cement	SF	FRHA	Sand	Granite	Course Granite	S.P.
	-	kg/m^3^	kg/m^3^	kg/m^3^	kg/m^3^	kg/m^3^	kg/m^3^	kg/m^3^
Control	0.3	560	-	-	725	305	695	9.02
SF5%C	0.3	532	28	0	725	305	695	9.02
SF10%C	0.3	504	56	0	725	305	695	9.02
SF15%C	0.3	476	84	0	725	305	695	9.02
SF20%C	0.3	448	112	0	725	305	695	9.02
SF25%C	0.3	420	140	0	725	305	695	9.02
SF30%C	0.3	392	168	0	725	305	695	9.02
FRHA5%C	0.3	532	0	28	725	305	695	9.02
FRHA10%C	0.3	504	0	56	725	305	695	9.02
FRHA15%C	0.3	476	0	84	725	305	695	9.02
FRHA20%C	0.3	448	0	112	725	305	695	9.02
FRHA25%C	0.3	420	0	140	725	305	695	9.02
FRHA30%C	0.3	392	0	168	725	305	695	9.02

Note: S.P.: Superplasticizer.

**Table 2:** Chemical compositions of cement, SF, and RHA.

Chemical Composition (%)	Cement	SF	RHA
SiO_2_	22.52	94	82.9
Al_2_O_3_	5.80	0.21	0.29
Fe_2_O_3_	3.52	0.09	1.83
SO_3_	2.54	-	1.34
CaO	62.08	0.12	2.29
MgO	1.55	0.33	0.93
Na_2_O	0.05	-	0.06
K_2_O	0.56	0.38	6.82
L.O.I.	0.94	1.2	1.5

Note: L.O.I.: Loss on ignition.

**Table 3:** Physical properties of cement, SF, CRHA, and FRHA.

Physical Properties	Cement	SF	CRHA	FRHA
Specific gravity	3.12	2.8	2.32	2.61
Surface area (m^2^/g)	0.34 (Blain)	21.08 (BET)	19.4 (BET)	23.6 (BET)
Mean particle size, d_50_ (μm)	12	5.11	9.49	5.69

Note: CRHA: RHA with 5 min grinding; and FRHA: RHA with 30 min grinding.

**Table 4:** Weight losses of pastes at the ages of 1, 3, 7 and 28 days.

Paste Specimen	Weight Loss (%)			Weight Loss with Respect to the Total Weight Loss (%)		
	Stage 1	Stage 2	Stage 3	Stage 1	Stage 2	Stage 3
Control-1d	3.16	2.16	2.47	40.57	27.73	31.70
Control-3d	4.15	2.88	2.39	44.09	30.53	25.37
Control-7d	4.96	3.88	2.26	44.70	34.95	20.35
Control-28d	5.21	3.95	2.50	44.69	33.85	21.46
SFP-1d	3.02	1.02	2.44	46.59	15.73	37.68
SFP-3d	4.62	2.97	1.93	48.51	31.24	20.25
SFP-7d	5.13	3.13	1.92	50.40	30.73	18.87
SFP-28d	5.33	3.24	1.87	51.04	31.04	17.92
FRHAP-1d	4.42	2.74	2.84	44.21	27.43	28.36
FRHAP-3d	5.55	3.00	3.04	47.89	25.89	26.22
FRHAP-7d	5.33	2.94	2.42	49.85	27.49	22.66
FRHAP-28d	5.51	3.17	2.37	49.90	28.67	21.43

Note: SFP: paste incorporating SF; and FRHAP: paste incorporating 30 min grinding RHA.

**Table 5:** Slump, compressive strength, and splitting tensile strength of concretes.

Concrete ID	Slump (mm)	Compressive Strength (MPa)				Splitting Tensile Strength (MPa)
		7d	14d	28d	90d	28d
Control	85	75.06	83.60	86.81	92.52	58.93
SF5%C	95	83.12	86.94	91.33	99.56	62.78
SF10%C	55	93.11	98.54	105.82	109.69	69.87
SF15%C	45	98.33	100.50	101.62	103.22	65.75
SF20%C	35	93.86	98.13	101.48	102.72	65.43
SF25%C	30	93.30	97.62	100.25	101.46	64.63
SF30%C	25	91.15	94.23	95.88	98.31	62.62
FRHA5%C	125	78.93	89.04	93.62	99.51	63.39
FRHA10%C	100	97.63	99.97	101.97	106.88	68.09
FRHA15%C	80	83.72	88.35	97.00	102.19	65.09
FRHA20%C	48	82.02	85.18	88.67	91.88	58.53
FRHA25%C	15	79.25	80.13	82.13	88.03	56.07
FRHA30%C	15	74.85	78.95	80.90	81.91	52.18

**Table 6:** Limit values according to ASTM C1202 [35].

Charge Passed (Coulombs)	Chloride Permeability
>4000	High
2000–4000	Moderate
1000–2000	Low
100–1000	Very low
<100	Negligible

**Table 7:** Coulomb charges of concretes at 28 days.

Concrete Specimens	Coulomb Charges of Concretes (C)	Evaluation of Concrete According to ASTM C1202 [35]
Control	528.67	Very low
SF5%C	493.28	Very low
SF10%C	212.44	Very low
SF15%C	198.36	Very low
SF20%C	188.63	Very low
SF25%C	273.28	Very low
SF30%C	292.6	Very low
FRHA5%C	478.69	Very low
FRHA10%C	327.67	Very low
FRHA15%C	393.2	Very low
FRHA20%C	567.44	Very low
FRHA25%C	1023.58	Low
FRHA30%C	2088.63	Moderate
